# Critical insights for intensivists on Guillain-Barré syndrome

**DOI:** 10.1186/s13613-025-01464-w

**Published:** 2025-05-21

**Authors:** Nicolas Weiss, Clémence Marois, Loic Le Guennec, Benjamin Rohaut, Sophie Demeret

**Affiliations:** 1 Département de neurologie, Service de Médecine Intensive Réanimation à orientation neurologique, Sorbonne Université, AP-HP.Sorbonne Université, Hôpital de la Pitié-Salpêtrière, 47-83, boulevard de l’hôpital, Paris, 75013 France; 2 Brain Liver Pitié-Salpêtrière (BLIPS) Study Group, Centre de recherche Saint- Antoine, Maladies métaboliques, biliaires et fibro-inflammatoire du foie, Institute of Cardiometabolism and Nutrition (ICAN), INSERM UMR_S 938, Paris, France; 3 Groupe de Recherche Clinique en REanimation et Soins intensifs du Patient en Insuffisance Respiratoire aiguE (GRC-RESPIRE) Sorbonne Université, Paris, France; 4 Institut Cochin, Leukemia and Niche Dynamics Laboratory, Université Paris Cité, INSERM, CNRS, Paris, France; 5 Paris Brain Institute - ICM, Inserm, CNRS, PICNIC-Lab, Paris, France

**Keywords:** Guillain-Barré syndrome, Miller-Fisher, Acute polyradiculoneuritis, Bickerstaff encephalitis

## Abstract

Guillain-Barré Syndrome (GBS) is a leading cause of acute flaccid tetraplegia worldwide, with an incidence of 1–2 cases per 100,000 people per year. Characterized by an immune-mediated polyneuropathy, GBS often follows infections or immunological triggers, including vaccinations. The syndrome is classified into three main subtypes based on electrophysiological findings: acute inflammatory demyelinating polyneuropathy (AIDP), acute motor axonal neuropathy (AMAN), and acute motor sensory axonal neuropathy (AMSAN). The pathophysiology of GBS involves molecular mimicry between microbial antigens and nerve structures, particularly affecting gangliosides and myelin proteins. Diagnosis primarily relies on clinical history, with lumbar puncture and electroneuromyogram used to confirm and differentiate subtypes. Treatment includes intravenous immunoglobulins or therapeutic plasma exchange associated with symptomatic treatment, especially mechanical ventilation if needed. Prognosis is generally favorable with a low mortality rate (< 5%) overall, but neurological sequelae can occur. Current research continues to explore novel therapeutic approaches, including complement-targeted therapies. Despite advancements, progress in specific treatments has been limited, and ongoing evaluation of potential biomarkers such as neurofilament light chains may enhance prognosis prediction and management strategies.

## Introduction

The Guillain-Barré syndrome (GBS) is the leading cause of acute flaccid tetraplegia worldwide, with an incidence of approximately 1–2 cases per 100,000 inhabitants per year [1–3]. The incidence is slightly higher in men than in women (sex ratio of 1.5) and increases with age, although it can occur at any age [1, 4]. GBS is an immune-mediated polyneuropathy often triggered by an infection or another immunological event, such as vaccination. Diagnosis is primarily based on anamnesis and clinical evaluation. Additional tests, including lumbar puncture and electroneuromyogram (ENMG), can often remain normal in the early stages and are generally used to rule out other differential diagnoses. Admission to the ICU is necessary in cases of respiratory failure (30% of cases, with 20% needing mechanical ventilation), impaired swallowing or dysautonomia (10% of cases) [2]. Treatment options include intravenous immunoglobulins or therapeutic plasma exchange, alongside supportive care. The long-term prognosis is generally favorable, with a mortality rate of less than 5%, although neurological sequelae can occasionally occur.

Although this syndrome may have been partially described in 1859 by Landry as “ascending paralysis,” it was definitively characterized as a distinct nosological entity in 1916 by Guillain, Barré, and Strohl [5, 6]. Their description included the clinical presentation, the biochemical feature of albumin-cytological dissociation, and electrophysiological findings, distinguishing GBS from other neuropathies, notably infectious neuropathies such as acute anterior poliomyelitis. GBS is classically divided into three types: acute inflammatory demyelinating polyneuropathy (AIDP), acute motor axonal neuropathy (AMAN), and acute motor sensory axonal neuropathy (AMSAN) [1, 2].

In this narrative review, we will successively discuss the triggering events of GBS, its pathophysiology, the classical clinical presentation, its diagnosis, the treatment strategies, the complications that can occur and the outcome, especially in severe forms requiring ICU.

### Triggering events

**Fig. 1:**
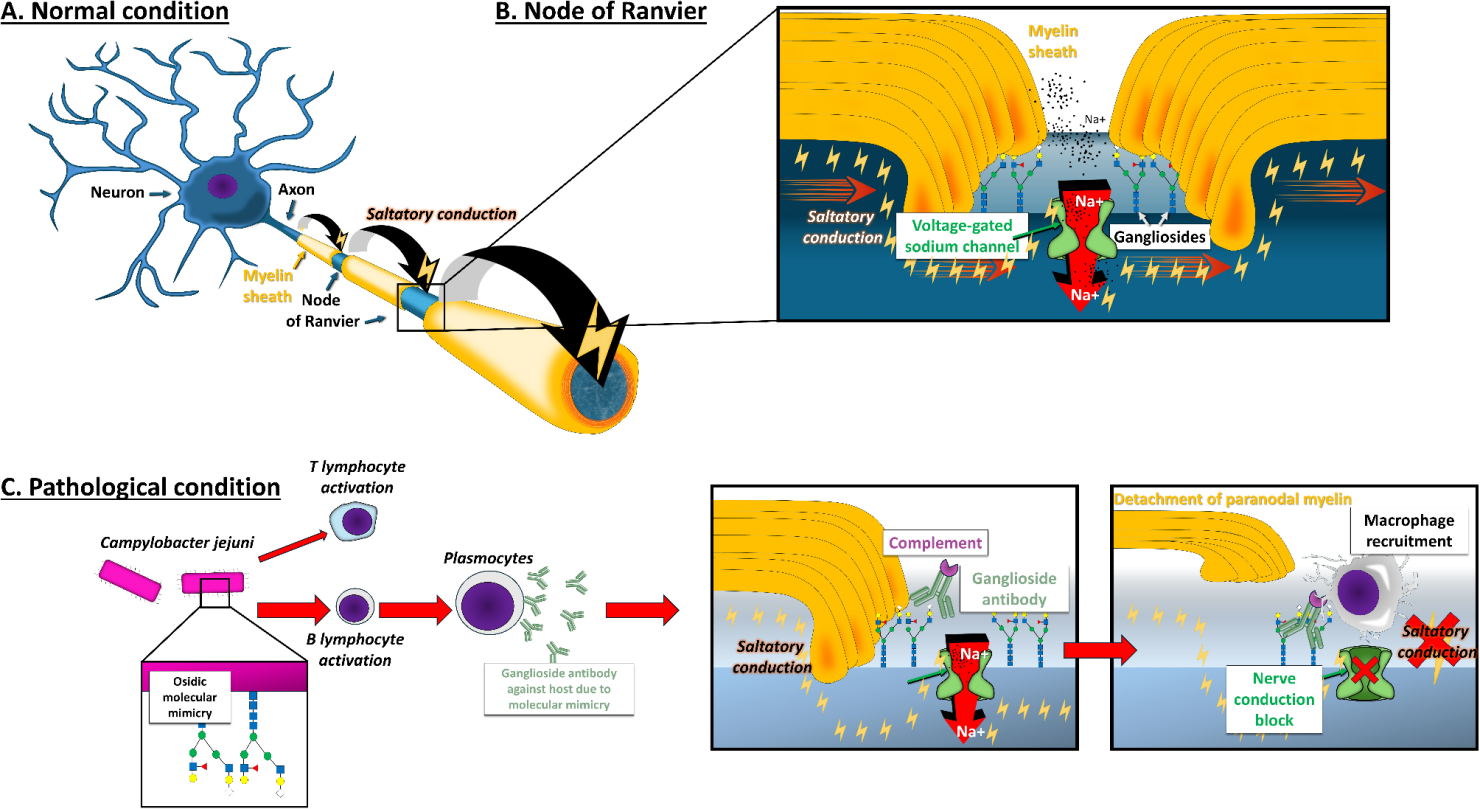
Pathophysiology of Guillain-Barré syndrome **A**, schematic view of the peripheral nerve showing the axon, the myelin sheath and the saltatory conduction from one node of Ranvier to another in normal condition; **B**, schematic view of the node of Ranvier showing voltage-gated sodium channel and gangliosides; **C**, immune system recognizes gangliosides expressed on the surface of *Campylobacter jejuni* and induces the production of gangliosides antibodies that are able to fix gangliosides expressed on the node of Ranvier; this correspond to what is coined as molecular mimicry. Antibodies fixation will lead to complement activation and then after macrophage recruitment

A few years after the initial description of GBS, physicians noticed a temporal link between symptom onset and recent infections, highlighting an abnormal immune response as a trigger [2, 7]. Indeed, two-thirds of GBS cases are preceded by an acute infectious event, as indicated by medical history and serological data (Fig. 1). *Campylobacter jejuni* is the most frequently identified infectious agent, particularly in Asia, where it accounts for about half of the cases [8–10]. Other bacterial triggers include *Mycoplasma pneumoniae* and *Haemophilus influenzae* [11], while viral triggers include cytomegalovirus, Epstein-Barr virus, Influenza-A virus [12], Hepatitis E virus [13], and arboviruses (Chikungunya, Dengue, and Zika viruses) [14, 15]. Whereas some associations have been proposed on case-series, six infectious agents have been conclusively associated with GBS through well-designed case-control studies: *Campylobacter jejuni*, cytomegalovirus, Epstein-Barr virus, Hepatitis E virus, *Mycoplasma pneumoniae*, and Zika virus [1, 2]. There have also been reports of GBS following SARS-CoV-2 infection [16].

More rarely GBS has been reported post-vaccination, particularly after the Semple type rabies vaccine and various influenza vaccines, with an incidence of approximately 1 case per 100,000 vaccinations. The risk of GBS following influenza vaccination is lower than after an influenza infection however [12]. Cases have also been reported after vaccinations against Human Papilloma virus, measles, mumps, rubella, *meningococcus* (tetravalent conjugate), and SARS-CoV-2 [1, 2]. However, the association with SARS-CoV-2 vaccines should be viewed in the context of a global mass vaccination campaign [16]. Globally, the protection offered by vaccination against infections that may cause GBS outweighs the risk of developing GBS following vaccination.

Other triggers include immunotherapies (anti-tumor necrosis factor, type I interferons, immune checkpoint inhibitors), chemotherapies (platin salts, brentuximab), surgery, or traumatic brain injury [17–19].

### Pathophysiology

GBS is classified into three subtypes based on electrophysiological findings: AIDP, AMAN and AMSAN. The pathophysiology of AMAN and AMSAN is the best understood, primarily attributed to molecular mimicry between infectious agent epitopes and nerve structures [20]. IgG antibodies targeting gangliosides, especially GM1 and GD1a at Ranvier nodes, have been identified in these subtypes (Fig. 1) [21–23]. In animal models, GBS-like symptoms can be induced by immunizing against these gangliosides, demonstrating a high homology between these nerve surface gangliosides and the glycan structures of various infectious agents [23]. This molecular mimicry hypothesis is well-documented, particularly for *Campylobacter jejuni* [9, 21–24]. Evidence suggests a similar mechanism for AIDP, with antibodies targeting myelin proteins and structures at the nodes of Ranvier, although the specific target antigens remain unknown [25]. The complement pathway, initiated by C1q binding, plays a crucial role, with C3 fragment deposition being more pronounced in GBS patients than in healthy controls [26].

Although a neuromuscular biopsy is not required for diagnosis, pathological data show complement deposition and macrophage infiltration in AIDP [27], AMSAN, and AMAN cases, with T lymphocytes commonly found in AIDP [26]. Myelin degradation in AIDP leads to conduction velocity reductions, while functional blockades characterize AMAN and AMSAN, disrupting nerve impulses due to Na+/K + ATPase blockade at Ranvier nodes [28, 29]. While molecular mimicry is a key factor in GBS pathophysiology, other factors, including genetic predispositions, remain largely underexplored [30–33]. Only a small percentage of *Campylobacter jejuni* infections lead to GBS, with certain strains linked to outbreaks [34, 35]. While most patients experience only one GBS episode, 2–5% may have recurrent episodes [36, 37]. Recently, antibodies targeting antigens at the node of Ranvier or the paranode have been implicated in GBS and in treatment-resistant chronic inflammatory demyelinating neuropathies (also called auto-immune nodo-paranodopathies) [38–40]. Interestingly, some of these patients initially presented with an acute clinical pattern closely resembling GBS [40]. This observation has led experts to speculate that certain cases of GBS, especially those responding poorly to treatment, may be associated with antibodies against proteins such as neurofascin or contactin [41, 42].

Although most patients recover spontaneously, irreversible damage can occur due to secondary axonal degeneration, resulting from persistent blockades or prolonged axonal denudation [43, 44].

### Clinical presentation

#### Classical presentation

GBS typically presents as a rapidly progressing, ascending symmetrical sensorimotor deficit [45]. It evolves through three distinct phases: progression, plateau, and recovery. In its classic form, patients often first experience with sensory disturbance (paresthesia, ataxia, radicular pain) in the lower limbs, which gradually ascends to the upper limbs over days—rarely hours—followed closely by motor deficits [1, 2, 45]. Initially, tendon reflexes may be present but will progressively decrease and eventually disappear (Table 1). Radicular pain and severe lumbar pain are common and can complicate the initial diagnosis, sometimes appearing as the first signs of the disease. The progression phase lasts, by the definition, less than four weeks, typically less than two weeks in 80% of cases [1, 2, 46]. This timing enables to distinguish GBS from other forms of subacute or chronic neuropathies for which an extensive work-up is mandatory [42, 47]. At the peak of the disease, motor symptoms can lead to tetraplegia, as well as paralysis of the facial, pharyngeal, and laryngeal muscles, resulting in impaired swallowing and aspiration. Involvement of the respiratory muscles can lead to respiratory failure, necessitating mechanical ventilation [1, 2, 46]. Dysautonomia, characterized by blood pressure and pulse lability, is also common. Patients may experience cardiac arrhythmias, blood pressure instability, urinary retention, or functional ileus [48, 49]. Dysautonomia is more frequent in severe cases, but it can occasionally present as a primary feature with minimal sensorimotor impairment.

**Table 1:** Guillain-Barré syndrome diagnostic criteria according to Brighton’s collaboration group. According Shahrizaila et al. Lancet 2021

	Brighton Collaboration (level of diagnostic certainty)			
	Level 1 (highest)	Level 2	Level 3	Level 4 (lowest)
**Clinical features**				
Bilateral and flaccid weakness of limbs	Yes	Yes	Yes	
Decreased or absent deep tendon reflexes	Yes	Yes	Yes	
Absence of alternative diagnosis	Yes	Yes	Yes	Yes
**Additional clinical features**				
Monophasic course, time between onset and plateau 12 h to 28 days	Yes	Yes	Yes	
Relative symmetry				
Mild sensory symptoms or signs				
Progress (usually after 2–4 weeks of plateau)				
Cranial nerve involvement (facial, bulbar and oculomotor)				
Autonomic dysfunction				
Absence of fever at the onset of neuritic symptoms				
**CSF analysis**				
CSF white cells count < 50/microL (usually < 10)	Yes	Yes*		
CSF protein raised (after week 1)	Yes	Yes*		
**Nerve conduction studies**				
Consistent with conduction slowing and block	Yes	Yes*		

Abbreviations: CSF, cerebrospinal fluid, * needed to have definite GBS

Overall, 20% of patients require mechanical ventilation, and 10–20% experience significant dysautonomia during the acute phase. Some patients endure severe pain that is difficult to manage despite multiple analgesic treatments [50], while others may primarily experience ataxia.

The plateau phase follows, during which symptoms stabilize and can last from a few days to several weeks, or even months in the most severe cases, such as those in ICU with a severe tetraplegia or a locked-in syndrome [43, 44, 51, 52]. The recovery phase follows, which can extend over several months.

#### Atypical presentation

**Fig. 2:**
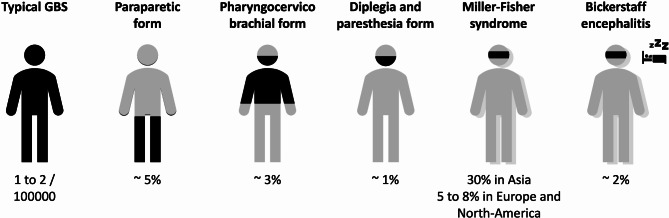
Different forms of Guillain-Barré syndrome Black areas represent localization of the neurological symptoms. The blurred representation stands for ataxia. The bed represents altered consciousness

Atypical presentations of GBS have been described, as reviewed by Yuki and Wakerley [53–58] (Fig. 2). The most common variant is Miller-Fisher syndrome, characterized by ophthalmoplegia, ataxia, and areflexia [59, 60]. This variant is considered a specific form of AMAN and is often associated with positive anti-GQ1b antiganglioside antibodies. Miller-Fisher syndrome can progress to a more typical GBS presentation, with symptoms descending from the initial signs to tetraplegia. Bickerstaff’s brainstem encephalitis, which combines GBS-like symptoms with altered consciousness, is a very rare entity [54, 61]. It may result from the direct action of anti-GQ1b antibodies on the ascending reticular activating system in the brainstem. When a GBS patient exhibits altered consciousness, more common complications, such as hyponatremia or posterior reversible encephalopathy syndrome secondary to extreme blood pressure fluctuation in the context of dysautonomia should be considered (see below) [49].

### Diagnosis

The diagnosis of GBS is primarily clinical, with lumbar puncture and ENMG used to confirm the diagnosis and exclude differential diagnoses (Table 2). The first set of diagnostic criteria was proposed by the National Institute of Neurological Disorders and Stroke (NINDS) in 1978 [62]. In 2011, an international committee of experts convened in Brighton to update these criteria, aiming to standardize epidemiological and pharmacovigilance studies (Table 1) [63]. Notably, neither the NINDS nor the Brighton criteria require additional diagnostic tests for a diagnosis of “probable GBS”. However, the Brighton criteria include lumbar puncture and ENMG results for confirming a diagnosis of “definite GBS”.

**Table 2:** Differential diagnosis of Guillain-Barré syndrome and its atypical forms

Guillain-Barré syndrome
***Transverse myelitis***
* Mycoplasma pneumoniae*
* Herpes simplex virus − 1 and − 2*,* cytomegalovirus*,* Epstein-Barr virus*,* varicella zoster virus*
Post-infectious myelitis
Initial manifestation of demyelinating disease (multiple sclerosis, neuromyelitis optica, etc.)
Nitrogen protoxide (therapeutic and recreational use)
***Spinal cord lesion***
Spinal compression (discal protrusion, epidural abscess or hematoma)
Anterior spinal cord artery occlusion
***Acute***,*** subacute and chronic neuropathies***
Infections (Diphteria, West-Nile virus, HIV, tick paralysis, Lyme disease, etc.)
Toxins or fish consumption (tetrodotoxine, lead, thallium, arsenic, etc.)
Drugs (cisplatine, brentuximab, etc.) *
Carential neuropathies (B1, B9, B12 vitamins)
Diabetes mellitus neuropathy
Hematological malignancies (MGUS, POEMS syndrome, CANOMAD, etc.)
Sarcoïdosis
Porphyria
Acute onset CIDP and auto-immune nodopathies (anti-neurofascein 155, anti-contactin-1 and probably anti-Caspr1 and anti-pan-neurofascein) ^$^
***Anterior horn cells disease / lower motor neuron syndrome***
Poliomyelitis, non-poliomyelitis enteroviruses (*enterovirus* 71), West-Nile virus
* Herpes simplex virus − 1 and − 2*,* cytomegalovirus*,* Epstein-Barr virus*, varicella zoster virus *
Rabies, HIV
***Neuromuscular junction disease***
Myasthenia gravis
Lambert-Eaton syndrome
Botulism (and adverse effect of therapeutic use of toxin botulinium)
***ICU acquired weakness (neuropathy)***
***Muscles diseases***
Acute myositis
Periodic paralysis and electrolytical disturbances (hypokaliemia, hypophosphatemia, hypermagnesemia, etc.)
Polymyositis and dermatomyositis
***Mitochondrial diseases***
***Somatoform diseases***
**Miller-Fisher syndrome and Bickerstaff encephalitis**
Myasthenia gravis
Brainstem stroke
Diphteria neuropathy
Botulism (and adverse effect of therapeutic use of toxin botulinium)
Rhombencephalitis
Infectious (listeria spp, tuberculosis, brucellosis, Lyme disease, *virus herpes simplex − 1 et -2*,* Epstein-Barr virus*, JC virus, toxoplasma spp, cryptococcosis, SARS-CoV2)
Inflammatory (multiple sclerosis, neuromyelitis optica, sarcoïdosis, Behçet disease, neurolupus, etc.)
Tumors (lymphoma, etc.) and paraneoplastic syndromes
Meningitis (inflammatory, infectious, carcinomatous and lymphoma)
Gayet-Wernicke encephalopathy

* could also represent triggers of GBS. ^$^ these nosological entities have been described very recently and their detailed clinical spectrum is not entirely known

Abbreviations: CANOMAD, chronic ataxic neuropathy, ophthalmoplegia, M-protein, cold agglutinins, and disialosyl antibodies (GD1b and GQ1b among others); Caspr1, contactin-associated protein 1; CIDP, chronic inflammatory demyelinating neuropathy; JC virus, John Cunningham virus; HIV, Human immunodeficiency virus; MGUS, monoclonal gammopathy of undetermined significance; POEMS, polyradiculoneuropathy, organomegalia, endocrinopathies, M component and skin; SARS-CoV2, severe acute respiratory syndrome coronavirus 2

#### Lumbar puncture

The primary purpose of a lumbar puncture in GBS evaluation is to exclude other potential diagnoses (e.g. Lyme disease, or varicella-zoster virus infection and other infections mainly). In the absence of conditions like diabetes mellitus or spinal compression, the presence of albumin-cytological dissociation—characterized by elevated protein levels with fewer than 10 cells per µL—is highly indicative of an inflammatory polyradiculitis [1, 2, 5, 45]. However, albumin-cytological dissociation may be absent in up to 50% of cases during the first week of symptom onset and in 10–30% of cases during the second week. While a mild cellular response can occasionally occur, cell counts rarely exceed 50 cells per µL. A pleocytosis between 10 and 50 cells per µL should prompt further investigation, as it may suggest an alternative diagnosis (e.g. infectious polyradiculonevritis mainly) (Table 2) [42, 46]. A lumbar puncture is particularly crucial when bilateral facial palsy or a cutaneous vesicular eruption is present, as these symptoms may indicate Lyme disease or varicella-zoster virus infection. If the CSF analysis is normal, it is not necessary to repeat it if the diagnosis is otherwise confirmed.

#### Electroneuromyogram

Like lumbar puncture, ENMG can initially yield normal results if performed early after symptom onset (in the first week). However, when abnormal, ENMG can confirm the diagnosis of GBS and help distinguish between its subtypes: AIDP, AMAN, and AMSAN [1, 2, 45, 64, 65]. Nerve conduction abnormalities are most pronounced around two weeks after symptom onset. Thus, if an ENMG has been performed in the first week and is normal a second one should be performed about one week later.

Typically, ENMG should be conducted on all four limbs, examining at least four different motor nerves and three sensory nerves, along with H reflexes and F waves. In AIDP, ENMG findings may include increased distal motor latencies, decreased conduction velocities, prolonged F wave latencies, temporal dispersion, and conduction block [29]. In AMAN, reduced amplitudes of motor waves are observed, while AMSAN presents with decreased amplitudes in both motor and sensory waves. Some cases of AMAN and AMSAN may also exhibit conduction block [28]. The ENMG is important to perform for diagnostic purposes and to rule out differential diagnoses. However, its performance should not delay the initiation of immunomodulatory treatment in case of clinical suspicion. It is often beneficial to perform ENMG twice to accurately differentiate between these subtypes, as initial testing can sometimes be inconclusive or fail to correctly classify the subtype [64, 66]. EMG may also be useful for prognostic purposes [119]. Nevertheless, in ICU, ENMG can be compromised by artefacts that can preclude a correct analysis.

#### Other exams

Apart from EMG and lumbar puncture, the most important examination is probably a spinal cord MRI in cases of suspected spinal cord lesions. The measurement of anti-ganglioside antibodies has limited diagnostic value for several reasons: the variability in analysis kits and their sensitivity, the delay of several weeks before results are available, and the low positivity rate observed in Europe [42]. However, in specific forms like Miller-Fisher syndrome, their presence is a significant diagnostic clue. Similarly, while infectious disease serologies can provide insights into the epidemiology of GBS, they are not crucial for diagnosis. Other microbiological techniques, such as cultures or PCR, are less useful given the post-infectious nature of the disease.

A minimal etiological work-up, as recommended for neuropathy, could include the following: a complete blood count, electrolytes with creatinine, HbA1c, B12 and folate levels, HIV serology, and serum protein electrophoresis and immunofixation. In cases of atypical presentation or nitrous oxide consumption, measuring homocysteine and methylmalonic acid levels can be useful [67, 68].

In certain situations, particularly with asymmetrical presentation or very acute onset (< 12 h), a cerebral or spinal MRI may be necessary to rule out a stroke. Recently, studies have suggested that nerve sonography could help in the diagnosis, as GBS patients often show enlarged nerve roots and trunks compared to controls [69, 70]. Although still preliminary, this technique could be particularly useful in the ICU. Additionally, elevated levels of neurofilament light chains have been observed in GBS patients, with their increase potentially correlating with disease severity and prognosis [71–73].

#### Differential diagnosis

The main differential diagnoses of GBS include infectious and inflammatory myelitis and infectious myeloradiculitis (Table 2). The presence of a sensory level and early bladder sphincter dissynergia even in the absence of a pyramidal syndrome should raise suspicion of a spinal cord lesion. Infectious myeloradiculitis caused by varicella-zoster virus or Lyme disease can present with similar symptoms. Vesicular lesions in specific dermatomes for varicella-zoster virus or facial diplegia in Lyme disease are highly suggestive. A lumbar puncture can help establish the correct diagnosis. Acute inflammatory neuropathy can also occur in the context of HIV seroconversion, but these cases are typically subacute, developing over more than four weeks and often associated with meningitis on lumbar puncture.

Some inflammatory neuropathies linked to systemic diseases, malignant hemopathies, or sarcoidosis can present acutely. An etiological work-up and thorough anamnesis are crucial for accurate diagnosis. A history of psychiatric symptoms, depression, or abdominal pain, especially if accompanied by dark urine, may suggest porphyria in cases of neuropathy with axonal impairment. Brainstem stroke is generally not a true differential diagnosis, as the mode of onset—sudden versus progressive—and brain MRI findings can easily differentiate it.

Occasionally, certain forms of diabetic neuropathies or alcoholic neuropathies can present acutely and mimic GBS. Additional differential diagnoses are listed in Table 2.

About 5% of patients initially diagnosed with GBS are finally diagnosed as having acute-onset chronic inflammatory demyelinating polyradiculoneuropathy [42, 47] (Table 3). This distinction is important since the treatment strategy slightly differs (repeated IV-Ig administration or use of corticosteroids). A very few patients diagnosed initially as GBS and responding poorly to treatment IV-Ig or TPE may have auto-immune para-nodopathy that constitute a recently described disease where antibodies directed against nodal or paranodal antigens (neurofascein 155, Caspr-1 or contactin) have been detected [39, 40]. Those antibodies might be look for in case of poor treatment response (see treatment section).

**Table 3:** Suspicious features for an acute onset chronic demyelinating inflammatory polyneuropathy rather than a GBS

Major features	Minor features
Progression > 8 weeks	No facial or bulbar weakness
More than 3 treatment related fluctuations	No respiratory weakness
Slower progression (possibly > 2 weeks from onset to nadir)	No preceding infection
	Absence of IgG anti-ganglioside antibodies
	Marked sensory abnormalities (including ataxia)
	Ultrasound evidence of widespread peripheral nerve enlargement
	Early significant reduction in motor nerve conduction velocity
Antibodies against nodal–paranodal antigens should be tested (important for treatment options)	

### Complications

#### Dysautonomia

Depending on the criteria used for diagnosis, between 10% and 50% of GBS patients experience dysautonomia due to autonomic nervous system involvement [48, 49, 74, 75]. However, it is crucial to rule out other causes of hypotension or cardiac arrhythmias, such as sepsis secondary to nosocomial infection, bleeding, or pulmonary embolism, before diagnosing dysautonomia.

Symptoms of dysautonomia include blood pressure fluctuations, cardiac arrhythmias, ileus, and urinary retention. The most common symptoms are sinus tachycardia, elevated blood pressure, and blood pressure instability [49, 74]. Unfortunately, there is no consensus definition of dysautonomia in GBS, and no specific recommendations on how and when to assess it. Some authors suggest diagnosing dysautonomia if systolic blood pressure varies by more than 85 mmHg within the same day without any confounding cause. This threshold is considered more sensitive than the previously proposed 40 or 50 mmHg daily variation [74]. Patients with blood pressure instability and tachycardia may be at a higher risk of cardiac arrhythmias, especially asystole. Although some data suggest that dysautonomia can occur in mild forms of GBS, the risk appears more prominent in severe cases presenting with tetraplegia, respiratory impairment, and swallowing difficulties during the ascending or plateau phase [74, 75].

23% of GBS patients have a heart rate above 100 beats per minute, rising to 75% among those requiring mechanical ventilation [75]. Even minimal stimulation can trigger severe bradycardia in these patients. In the most severe cases, mild activities such as tracheal suctioning, intubation, mouth care, or bathing can provoke severe dysautonomia symptoms, particularly bradycardia. Some teams recommend having 1 mg of IV atropine readily available for severe dysautonomia. Outside of severe bradycardia, conservative management is generally preferred. It is risky to use antihypertensive drugs or antiarrhythmics unless absolutely necessary. A pacemaker is rarely indicated. Ileus is frequently problematic and can lead to functional bowel obstruction. Maintaining normal potassium levels and avoiding sedatives is advisable. Posterior reversible encephalopathy syndrome due to extreme blood pressure fluctuation is possible (7% of dysautonomic patients), and may be exacerbated by IV immunoglobulin treatment [49]. Takotsubo cardiomyopathy is another rare life-threatening complication of GBS, probably enhanced by dysautonomia, secondary to the activation of the sympathetic nervous system and an increase in catecholamines [120].

#### Salt wasting syndrome or inappropriate secretion of antidiuretic hormone

The exact nature of hyponatremia in GBS remains debated. Some experts suggest that hyponatremia is primarily due to salt-wasting syndrome [76–78]. A recent pathophysiological study proposed that this could be linked to altered adrenal gland autoregulation due to autonomic nervous system dysfunction, although further confirmation is needed [78]. IV immunoglobulin can also contribute to hyponatremia by increasing serum protein levels, leading to pseudohyponatremia. Clinically, hyponatremia typically responds to the administration of sodium chloride, either intravenously or orally, with the dosage adjusted based on urinary sodium losses. It is important to note that hyponatremia can exacerbate neurological symptoms.

#### Vivid Dreams

GBS patients can occasionally experience altered sleep with depersonalization symptoms, described as vivid dreams, which may be misdiagnosed as delirium [79]. These symptoms may be underdiagnosed, especially in severe patients on MV, where communication is impaired. In cases where such complaints arise in patients not yet on MV, consideration should be given to transferring them to the ICU, as these symptoms could be associated with dysautonomia and a more severe illness.

### Treatment strategies in Guillain-Barré syndrome

#### Specific treatments

Treatment strategies for GBS have been extensively documented and summarized in Cochrane reviews [80–82]. The European Academy of Neurology recently published guidelines [42]. The standard of care involves the administration of immunotherapy, either therapeutic plasma exchange (TPE) or intravenous immunoglobulins (IV-Ig) for patients still in the ascending phase, ideally in the first 2 weeks of symptom’s onset (Table 4) [42]. TPE was the first validated as a treatment for GBS in the 1980s, with several randomized controlled trials (RCTs) demonstrating that it improved the proportion of patients able to walk without aid at one month compared to placebo [80]. Whereas it has been shown that 6 TPE was not better than 4 even in severe patients that were mechanically ventilated, it has been shown that 2 TPE was better than placebo in patients with mild GBS [83]. Studies showed also that using fresh frozen plasma as replacement fluid was not superior to using albumin and crystaloïds but was associated to more side-effects [83, 84]. It should be noted that risk of bleeding, infection and severe cardiovascular instability are a classical counter-indication for TPE or should be at least discussed. Recent European Academy of Neurology guidelines recommend, if TPE is chosen, to perform 4 to 5 TPE over one to 2 weeks for a total exchanged volume of 12 to 15 L [42].

**Table 4:** Treatment strategies in Guillain-Barré syndrome

Specific GBS treatments		
Treatment	Modalities	Dosage
Therapeutic plasma exchange	4 to 5 sessions in the severe ICU forms Replacement solute: Albumin 5% (or fresh frozen plasma if altered blood coagulation, e.g. fibrinogen < 2 g/L)	One course of 12 to 15 L in 4 to 5 exchanges over 1–2 weeks
IV-immunoglobulins	Administration over 5 days (more rapid administration could be associated with more TRFs)	Total dosage of 2 g/kg (0.4 g/kg for 5 days) IV
Second course	Only in case of TRFs	Total dosage of 2 g/kg (0.4 g/kg for 5 days) IV
Switch of TPE to IV-Ig or IV-Ig to TPE	Not recommended (even if no clear improvement or deterioration occurs) In auto-immune nodopathies, TPE might be effective whereas IV-Ig are not	-
Corticosteroïds	Not recommended	-
**Symptomatic treatments**		
**Mechanical ventilation**: (see Fig. 2)		
**Pain management**: - Antiseizure medications used for neuropathic pain, particularly gabapentin and pregabalin or tricyclic antidepressants. Carbamazepin that has been tested is not preferred due to possible drug interaction. Paracetamol can be problematic due to possible mild liver enzyme elevation in the initial phase. The use of local anesthetics, especially lidocaine 5% plaster might be discussed.		
**Prevention of further complications**: - Thromboembolic events prevention - Early mobilization and appropriate positioning to prevent contractures, to avoid *equinus varus* and the development of osteoma - Bowel dysfunction close surveillance - Glucose level monitoring		

Abbreviations: Ht, hematocrit; IV, intravenous; IV-Ig, IV immunoglobulins; TPE, therapeutic plasma exchange; TRFs, treatment related fluctuations

In the 1990s, RCTs compared IV-Ig with TPE found no significant difference in the primary outcome of patients being able to walk without aid at one month [81]. The recommended dosage for patients unable to stand-up is 0.4 g per kg per day for 5 days [42]. A more rapid administration (2 days versus 5 days) could be associated to more treatment related fluctuations and should thus be discouraged [81, 85]. Recently, the Erasmus group in the Netherlands conducted a randomized, double-blinded, placebo-controlled trial to test the efficacy of a second course of IV-Ig, 2 g/kg administered over 5 days, administered 7 to 9 days after the initial course, in patients with a poor prognosis, assessed by a modified Erasmus Guillain-Barré syndrome Outcome Score ≥ 6 [86]. This trial, which included 93 patients, did not show any significant difference in the primary outcome, the Guillain-Barré syndrome disability score at 4 weeks post-inclusion, nor any outcome measure but the second course was associated with more frequent thromboembolic complications.

In current clinical practice, most GBS patients are treated with a single course of IV-Ig, as it can be administered by non-specialized teams and does not require the specific equipment needed for TPE. Nevertheless, TPE could be efficient in auto-immune para-nodopathies resistant to IV-Ig [87]. Some limited data suggest that IV-Ig could be associated with a lower duration of mechanical ventilation [88]. The environmental impact has, until now, nevertheless not be evaluated.

One recurrent discussion in ICU is whether a mechanically ventilated patients should be treated by a second course of IV-Ig or a switch from IV-Ig to TPE or from TPE to IV-Ig for insufficient or no response at one month. This question is insufficiently addressed today in the literature but the guidelines recommend against an alternative treatment (TPE or IV-Ig) as they do not recommend to administer IV-Ig immediately after TPE [42]. One RCT comparing TPE, IV-Ig and IV-Ig started after the last TPE did not found any difference in outcome [89] and as the efficacy of IV-Ig and TPE seems similar, the experts consider that the effect of TPE after IV-Ig has no reason to differ.

This former question is slightly different from the case of a patient that initially improved or stabilized with treatment, and who secondarily deteriorates with a neurological worsening. This condition is called treatment related fluctuations and might be present in 10% of GBS patients according to one study [47, 90]. These fluctuations might beneficiate from a re-treatment with either IV-Ig or TPE according to observational studies, i.e. no RCT available [42].

In patients with poor response to treatment, continuous worsening, or relapse after treatment, testing for antibodies against nodal–paranodal antigens might be valuable [39, 40, 42].

It should be noted that several well-designed studies have assessed the effectiveness of corticosteroids, administered either orally or intravenously. These studies, summarized by the Cochrane group, consistently found no beneficial effect [82]. Some prospective studies even suggested a potential harm with a worsening of symptoms when corticosteroids were used in this context. Consequently, corticosteroids are not recommended for the treatment of GBS [42].

#### Symptomatic treatments

In addition to specific treatments, it is crucial to manage respiratory failure, alleviate pain, dysautonomia and prevent decubitus complications in GBS patients.

i. *Mechanical Ventilation*.

- Need for mechanical ventilation.

Various studies have suggested that delayed use of mechanical ventilation (MV) in GBS was associated with increased morbidity, including aspiration pneumonia and cardio-respiratory arrest [91–93]. However, a recent monocentric randomized trial including 55 patients did not found any difference in the incidence of pneumonia between patients with early VM and those with delayed VM [94].

**Fig. 3:**
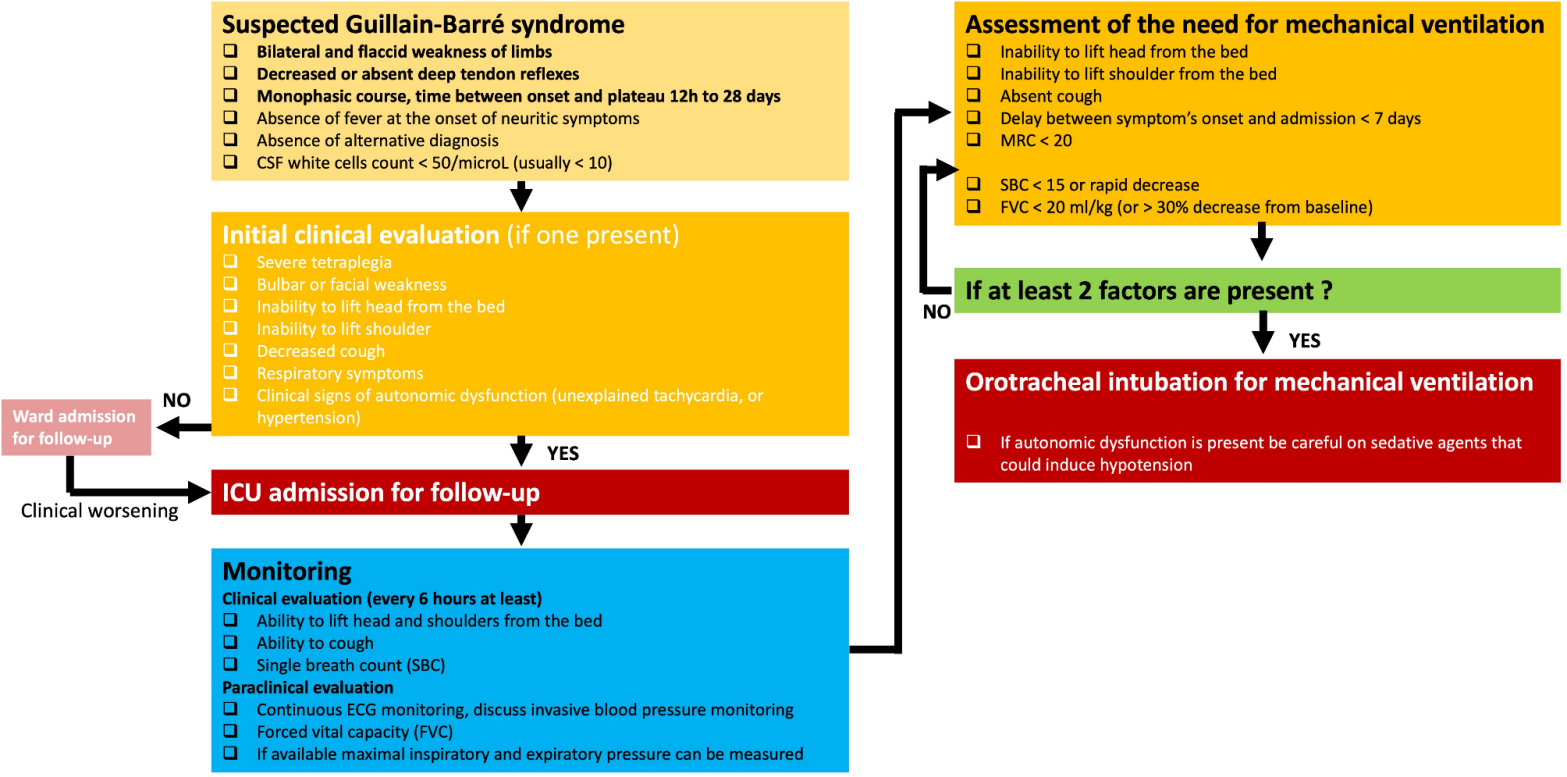
Initial management of Guillain-Barré syndrome Abbreviations: CSF, cerebrospinal fluid; FVC, forced vital capacity; MRC, medical research council motor score; SBC, single breath count

Although the criteria for initiating MV have long been based on expert opinion and small retrospective studies, a few larger-scale studies have established robust criteria. A large French retrospective study identified six independent predictive risk factors for the need for MV: inability to cough (OR 9.09), inability to abduct shoulder to horizontal (OR 2.99), neck flexion weakness (OR 4.34), GBS disability grade ≥ 4 (wheelchair bound or bedridden) (OR 2.53), a delay of less than 7 days between symptom onset and hospital admission (OR 2.51), and liver cytolysis (OR 2.09) (Table 5) [95]. The presence of four of these criteria was associated with an 85% risk of requiring MV. Additionally, the presence of dysautonomia has also been identified as a risk factor for requiring MV. The EGRIS (Erasmus GBS Respiratory Insufficiency Score) [96] and mEGRIS (modified EGRIS) scores [97] (Table 5), validated on an international cohort, assess the risk of respiratory failure within the first week after admission based on three factors: the time between disease onset and admission, bulbar involvement, and the MRC score [96–98]. Clinically, assessing the patient’s ability to lift their head off the bed and perform forced vital capacity is a common practice (Fig. 3) [99–101]. A single breath count (SBC), where the patient takes a maximal inspiration and counts without taking another breath, is sometimes used as an approximation [102]. Inability to lift the head and an apneic count below 15 have been proposed as good markers for the need for mechanical ventilation (Table 5). If forced vital capacity is measured at the bedside, a value below 20 ml/kg or a 50% decline in 24 h is considered a strong prognostic factor and indicates an already advanced diaphragmatic dysfunction and should lead to prompt intubation [42]. Pulse oximetry is rarely useful, as oxygen desaturation typically occurs late in restrictive respiratory insufficiency. Some electrophysiological data, such as the amplitude of the diaphragmatic muscle action potential, have been explored to predict the need for MV, but with rather disappointing results.

**Table 5:** Predictive criteria for the need of mechanical ventilation in Guillain-Barré syndrome

Erasmus Guillain-Barré Syndrome Respiratory Insufficiency (EGRIS) score		
**Parameters**	**Categories**	**Score**
Symptom’s onset to hospital admission delay	> 7 days	0
	4 to 7 days	1
	≤ 3 days	2
Facial or bulbar weakness at hospital admission	No	0
	Yes	1
Motor deficit at hospital admission (*Medical Research Council (MRC)* scoring^***+***^)	60 − 51	0
	50 − 41	1
	40 − 31	2
	30 − 21	3
	≤ 20	4
**Total score**		**0 to 7**
**Interpretation**: Probability of need for mechanical ventilation in the first week of hospitalization: - score of 0 to 2, 4% ; score of 3 to 4, 24% ; and score of 5 to 7, 65%		
^+^ Sum of MRC scoring of deltoïds, biceps, carpal radial extensors, iliopsoas, quadriceps and anterior tibial muscles.		
***Modified Erasmus Guillain-Barré Syndrome Respi*** ***ratory Insufficiency (mEGRIS) score***		
**Parameters**	**Categories**	**Score**
Bulbar weakness	Yes	5
	No	0
Symptom’s onset to hospital admission delay	0	7
	1	6
	2	5
	3	4
	4	3
	5	2
	6	1
	≥ 7	0
MRC score of nuchal flexion (0–5)	0	10
	1	8
	2	6
	3	4
	4	2
	5	0
MRC score of leg flexion (0–10)	0	10
	1	9
	2	8
	3	7
	4	6
	5	5
	6	4
	7	3
	8	2
	9	1
	10	0
**Total score**		**0 to 32**
**Interpretation**: Probability of need for mechanical ventilation at day 1, day 3 and day 7 according to a S shaped curve. Briefly, the probability to be ventilated at day 7 is: − 5% for a score of 8, 25% for a score of 16 and 65% for a score of 22.		
***Bedside clinical evaluation (5 criteria)***		
Neck flexion weakness		
Inability to abduct shoulder to horizontal		
Inability to cough		
Inability to stand-up (GBS disability grade ≥ 4, wheelchair bound or bedridden)		
A delay of less than 7 days between symptom onset and hospital admission		
Liver cytolysis		
**Interpretation**: The presence of four of these criteria was associated with an 85% risk of requiring mechanical ventilation.		
**Other predictive parameters**		
Forced vital capacity < 20 mL/kg or rapid decrease (> 30% of baseline)		
Rapid decrease in single breath count or a value < 15		
Clinical markers of autonomic failure		
MIP < − 40 cmH2O and MEP of < + 30 cmH2O if performed		
Axonal damage at ENMG		

Abbreviations: ENMG, electroneuromyogram; GBS, Guillain-Barré syndrome; MEP, maximal expiratory pressure; MIP, maximal inspiratory pressure

**Table 6:** What’s new for the intensivist

**1.**	**Acute-onset chronic inflammatory demyelinating polyradiculoneuropathy** About 5% of patients initially diagnosed with GBS are finally diagnosed as having acute-onset chronic inflammatory demyelinating polyradiculoneuropathy. This distinction is important since the treatment strategy slightly differs (repeated IV-Ig administration or use of corticosteroids). The time from first neurological to maximal symptoms classically exceed 4 weeks
**2.**	**Auto-immune nodo-paranodopathies** Recently, antibodies targeting antigens at the node of Ranvier or the paranode have been implicated in GBS and in treatment-resistant chronic inflammatory demyelinating neuropathies (also called auto-immune nodo-paranodopathies). Interestingly, some of these patients initially presented with an acute clinical pattern closely resembling GBS. This observation has led experts to speculate that certain cases of GBS, especially those responding poorly to treatment, may be associated with antibodies directed against nodal or paranodal antigens (neurofascein 155, Caspr-1 or contactin)
**3.**	**Treatment related fluctuations** Some GBS patients that initially improved or stabilized with treatment can secondarily present a neurological worsening. This condition is called treatment related fluctuations and might be present in 10% of GBS patients. These fluctuations might beneficiate from a re-treatment with either IV-Ig or TPE according to observational studies. This condition should be differentiated from acute-onset chronic inflammatory demyelinating polyradiculoneuropathy
**4.**	**Nerve sonography** Nerve sonography could help in the diagnosis, as GBS patients often show enlarged nerve roots and trunks compared to controls
**5.**	**Neurofilament light chains** Elevated levels of neurofilament light chains have been observed in GBS patients and their elevation could represent a prognostic marker
**6.**	**Complement-targeted therapies** Strategies aimed at blocking complement activation such as eculizumab, which inhibits the cleavage of C5 into C5a and C5b and thus the final activation of the complement cascade, has been tested with inconclusive results in small RCTs. Blockade of the complement cascade before the final activation of the complement cascade might be another promising strategy.

For intubation, presence of significant dysautonomia should favor the use of non-hypotensive induction agents.

Non-invasive ventilation (NIV) is usually contraindicated in the presence of swallowing disorders, bilateral facial paralysis, or ineffective cough. NIV seems thus not appropriate in the GBS due to the lack of rapid improvement in respiratory function within a few hours. However, it can be used for preoxygenation.

b. Timing of tracheostomy.

The duration of MV is often prolonged in GBS with median MV times estimated between 21 and 28 days [93, 95–98]. The lack of foot flexion ability at the end of immune therapy was proposed as a good bedside predictor of prolonged duration of MV in one study [103]. In a study including 212 GBS patients hospitalized in ICU from 2001 to 2011, 22 (10%) required mechanical ventilation for more than two months, with an average time to decannulation from tracheostomy of three months [44]. Risk factors for prolonged ventilation include the severity of motor deficit, the severity of axonal involvement on ENMG, advanced age, and a history of lung disease [93, 100]. In the absence of strong data, it seems reasonable to consider a tracheostomy in a patient who remains ventilator-dependent after 2 weeks and has failed multiple weaning trials, as well as in a patient who has had at least one failed extubation. The advantages of tracheostomy in these conscious patients might include greater comfort, earlier and safer mobilization to a chair, improved oral hygiene, facilitated communication, and better assessment of swallowing and bulbar function during weaning [104]. The presence of a tracheostomy allows for gradual discontinuation of respiratory assistance while keeping the cannula in place until adequate secretion clearance is restored. Dysphagia due to persistent laryngeal sensory deficit could contribute to delayed decannulation [105].

c. Mechanical ventilation weaning.

**Fig. 4:**
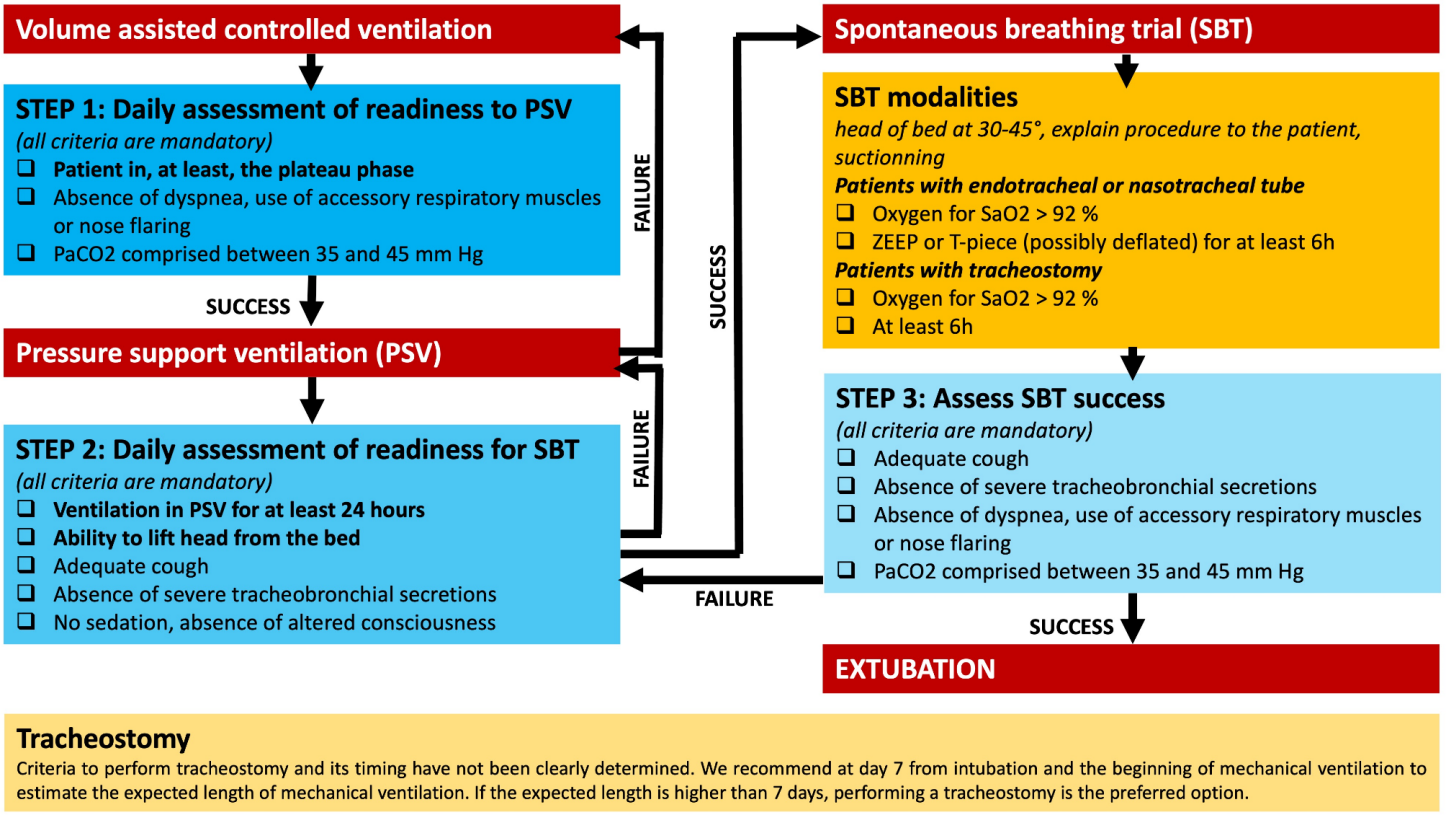
Proposed mechanical ventilation weaning algorithm Abbreviations: HR, heart rate; P/F, PaO2/FiO2 ratio; PEEP, Positive end expiratory pressure; PSV, Pressure support ventilation; RR, respiratory rate; SBP, systolic blood pressure; SBT, spontaneous breathing trial; ZEEP, zero end expiratory pressure

Weaning from ventilation should be considered once general clinical improvement has begun, usually after etiological treatment (intravenous immunoglobulins or TPE) are terminated (Fig. 4). Weaning modalities are primarily based on expert opinion, as studies are limited, often monocentric, retrospective, and small in size [100, 101, 106, 107] or GBS patients excluded [132]. It is generally accepted that pressure ventilation modes should be favored as soon as the patient can tolerate them, with a gradual decrease in inspiratory support levels. For patients with severe GBS, this may take several days or weeks. It is also important to note that these patients are at high risk for atelectasis, and some centers favor pressure ventilation during the day and volume ventilation at night.

The usual weaning criteria (hemodynamic, respiratory, and neurological) are necessary but not sufficient to predict successful extubation in neuromuscular patients. Several prerequisites are required for successful extubation and, therefore, the initiation of a weaning trial: effective cough, low secretions, absence of ongoing respiratory infection, and the ability to lift the head off the bed (Fig. 4).

Several studies have used forced vital capacity to guide weaning. These measurements are easily performed on recent ventilator models. Some studies suggest that SBTs can be considered when forced vital capacity exceeds 15 ml/kg or increases by 4 ml/kg from pre-intubation values [108]. Another study suggested starting weaning when forced vital capacity reached 7 ml/kg, with increasingly prolonged SBTs, and then extubation when forced vital capacity exceeded 15 ml/kg and the patient breathed freely for 24 consecutive hours. Some authors recommend taking pressure measurements into account and suggest performing SBTs only when maximal inspiratory pressure > -20 cmH2O and/or maximal expiratory pressure > + 40 cmH2O [42].

Progressive increase in the duration of SBTs over days and prolonged SBTs, lasting more than 2 h, have been proposed to better assess respiratory muscle fatigue [129–131]. However, the ideal duration of the trials is not determined, ranging from 2 to 24 h, with the average proposed duration being 6 to 8 h. SBTs are generally repeated, with progressively increasing duration.

There is no data on the preferred type of SBT. However, prolonged cuff-inflated T-piece SBTs carry a risk of tube obstruction, favoring the use of ZEEP. Once the inspiratory muscles can ensure normal ventilation (RR, Vt, EtCO2, P/F) during a prolonged cuff-inflated SBT, secretion drainage function might be assessed with progressively longer cuff-deflated T-piece SBTs, up to 6–8 h if necessary. This technique also tests bulbar dysfunction, albeit imperfectly. Extubation failure is often related to an inability to clear secretions, and a patient who cannot tolerate a deflated cuff and cannot clear their secretions is at high risk for extubation failure. Extubation failures have also been correlated with the presence of pneumonia or persistent dysautonomia.

The use of NIV or high-flow oxygen therapy after extubation has not yet been studied in this population but could be an interesting technique to consider a few hours after extubation in patients at risk of atelectasis.

ii. *Pain management*

Pain management can be challenging due to the involvement of sensory nerves, leading to significant pain, paresthesia, radiculalgia, and low back pain [50]. Treatment options are limited. A Cochrane review [109] summarized data from three RCTs involving 277 GBS patients. Although the results were negative, gabapentin may be slightly effective in the acute phase, and carbamazepine may help in the long-term. In common practice, antiseizure medications used for neuropathic pain, particularly gabapentin and pregabalin, are frequently used (Table 4). Paracetamol can be problematic due to possible mild liver enzyme elevation in the initial phase of GBS. Occasionally, level 2 or 3 analgesics are necessary, though results are inconsistent and they can trigger deleterious side-effects especially ileus. Local anesthetics, such as lidocaine 5% plaster, may be tried with a low risk of adverse effects [110]. To note that if dysautonomia is present some medications should be used with caution (e.g. tricyclic antidepressants such as amitriptyline).

iii. *Maintain effective communication and provide psychological support*

Maintaining effective communication is crucial for patient care and management. For patients with the most severe motor impairment, this can lead to a locked-in syndrome. Advanced communication interfaces that utilize visual tracking, subtle movement detectors, or various neurophysiological signals to control a computer can serve as essential communication aids. These devices enable patients to express their needs, symptoms, and concerns even when conventional communication is impossible, maintaining a connection with healthcare providers and family members, and reducing the psychological burden of their condition. In the context of GBS management in the ICU, these communication aids can also be invaluable for early detection of complications and for tailoring symptomatic treatment to the patient’s specific needs. It is also essential to screen for and treat psychological disorders early during hospitalization through a multidisciplinary approach [133]. Anxiety is indeed associated with weakness and bulbar dysfunction and with respiratory failure in these patients [121].

iv. *Prevention of further complications*

Preventing decubitus complications is essential. Proper patient positioning should prevent *equinus varus*, a deformity that can lead to a fixed position requiring tenotomy. Early mobilization and appropriate positioning by a physiotherapist are crucial to prevent contractures and the development of osteoma, although these measures are not specific to GBS. It is also important to control blood sugar levels with an appropriate insulin therapy protocol. Indeed, the occurrence of hyperglycemia is correlated with poorer outcomes upon discharge from intensive care in ventilated GBS patients [122]. Although there are no very robust studies, several studies suggest that exercise programmes improve physical outcomes such as functional mobility, cardiopulmonary function, isokinetic muscle strength, and work rate and reduce fatigue in patients with GBS. Thus, multidisciplinary rehabilitation is recommended but may be challenging in ICU because of limited patient participation [123, 124].

v. *Therapeutic perspectives*

No significant progress has been made in the treatment and management of GBS since the 1990s [42, 111]. Strategies aimed at blocking complement activation using eculizumab, which inhibits the cleavage of C5 into C5a and C5b and thus the final activation of the complement cascade, showed promise in animal studies [112]. However, these strategies have been tested in only two small phase 3 studies in humans [113, 114]. The first study included 8 patients, with 5 receiving eculizumab and 3 receiving a placebo. The second study involved 34 patients, with 23 in the eculizumab group and 11 in the placebo group. Unfortunately, both studies did not meet their primary endpoint of independent walking at one month. A recent phase 3 RCT comparing eculizumab as an add-on therapy to IV-Ig compared to placebo that included 57 patients with severe GBS (eculizumab, *n* = 37; placebo, *n* = 20) failed nevertheless to demonstrate any effect on primary or secondary outcomes [125]. Blockade of the complement cascade before the final activation of the complement cascade might be another strategy.

### Outcome

Despite the general perception of a “favourable outcome” in GBS, 16% of severe cases admitted to the ICU remain unable to walk independently at one year, and about 30% still experience chronic pain [1, 2, 115]. Only 50% of patients recover independent walking at six months with the established standard of care. Two third of GBS patients suffered complication during their ICU stay. Main complications are ventilator-associated pneumonia (between 30 and 78% of patients), acute respiratory distress syndrome (26%), septic shock (22–24%), ileus and/or bowel perforation (17%), pulmonary embolism (7%), gastrointestinal hemorrhage (7%), acute renal failure (4%) or complications of tracheostomy (4%) [126–128]. The overall mortality rate for GBS remains relatively low, around 3–5% mainly from ICU complications [58]. However, in a retrospective cohort, mortality was three times higher (6% versus 2%) among patients with dysautonomia [74].

The most important factor being associated with poor outcome in the literature is the abnormal mean amplitude of compound muscle action potential on ENMG [116, 117]. Others factors are: older age, time from onset of disease to hospitalization of 7 days or less, and need for ventilatory support. The mEGOS (modified Erasmus GBS Score) might be used to assess this prognosis. This score includes the age at onset of the disease, the presence of diarrhea preceding GBS, and the MRC score at day 7 of admission [118]. Elevated plasma levels of neurofilament light chains may serve as an early surrogate marker for long-term outcomes in the future [71, 72].

Recent guidelines from the neurocritical care society however suggest to consider the complete clinical condition and not only a single variable during prognostication [115]. For patients with prolonged ventilation, especially the elderly, an ethical discussion regarding the intensity of care may sometimes be necessary. In such cases, the patient’s wishes should be at the center of the discussions.

In addition to physical disabilities, these patients often suffer from psychological burdens, including frequent post-traumatic stress disorder (PTSD) even years after their ICU stay [44]. GBS constitute a significant economic burden, with prolonged ICU stays (often several months), extended rehabilitation periods, and ongoing financial support for specialized care and unemployment compensation.

There is a critical unmet medical need for improved management of the most severe GBS patients.

## Conclusion

GBS remains a significant clinical challenge due to its varied presentation, complex pathophysiology, and potential for severe complications. Although the prognosis is generally favorable with appropriate management, the potential for long-term neurological sequelae underscores the importance of early diagnosis and treatment. While immunotherapies like IV-Ig and TPE are the mainstays of treatment, recent advancements in understanding the disease’s molecular mechanisms offer hope for novel therapeutic approaches. However, current options for managing GBS complications, such as respiratory failure and dysautonomia, require meticulous care and tailored interventions. As research continues to unravel the intricacies of GBS, new insights into its etiology and pathophysiology may pave the way for improved patient outcomes and innovative therapies.

## Data Availability

Not applicable, review article.

## References

[1] Yuki N, Hartung HP. Guillain-Barré syndrome. N Engl J Med. 2012;366(24):2294–304. 22694000 10.1056/NEJMra1114525

[2] Willison HJ, Jacobs BC, van Doorn PA. Guillain-Barré syndrome. Lancet. 2016;388(10045):717–27. 26948435 10.1016/S0140-6736(16)00339-1

[3] Sejvar JJ, Baughman AL, Wise M, Morgan OW. Population incidence of Guillain-Barré syndrome: a systematic review and meta-analysis. Neuroepidemiology. 2011;36(2):123–33. 21422765 10.1159/000324710 PMC5703046

[4] Lu JL, Sheikh KA, Wu HS, Zhang J, Jiang ZF, Cornblath DR, et al. Physiologic-pathologic correlation in Guillain-Barré syndrome in children. Neurology. 2000;54(1):33–9. 10636122 10.1212/wnl.54.1.33

[5] Guillain G, Barré J, Strohl A. Sur Un syndromede radiculo-névrite avec hyperalbuminose du Liquide Céphalorachidien Sans réaction cellulaire. Remarques Sur les caractères cliniques et graphiques des réflexes tendineux. Bull et Mem de la Soc Méd des Hop de Paris. 1916;1462–70. 10400560

[6] Goodfellow JA, Willison HJ. Guillain-Barré syndrome: a century of progress. Nat Rev Neurol. 2016;12(12):723–31. 27857121 10.1038/nrneurol.2016.172

[7] Jacobs BC, Rothbarth PH, van der Meché FG, Herbrink P, Schmitz PI, de Klerk MA, et al. The spectrum of antecedent infections in Guillain-Barré syndrome: a case-control study. Neurology. 1998;51(4):1110–5. 9781538 10.1212/wnl.51.4.1110

[8] Ho TW, Willison HJ, Nachamkin I, Li CY, Veitch J, Ung H, et al. Anti-GD1a antibody is associated with axonal but not demyelinating forms of Guillain-Barré syndrome. Ann Neurol. 1999;45(2):168–73. 9989618 10.1002/1531-8249(199902)45:2<168::aid-ana6>3.0.co;2-6

[9] Huizinga R, van den Berg B, van Rijs W, Tio-Gillen AP, Fokkink WJR, Bakker-Jonges LE, et al. Innate immunity to Campylobacter jejuni in Guillain-Barré syndrome. Ann Neurol. 2015;78(3):343–54. 26017721 10.1002/ana.24442

[10] McCarthy N, Andersson Y, Jormanainen V, Gustavsson O, Giesecke J. The risk of Guillain-Barré syndrome following infection with Campylobacter jejuni. Epidemiol Infect. 1999;122(1):15–7. 10098780 10.1017/s0950268898001861 PMC2809582

[11] Mori M, Kuwabara S, Miyake M, Noda M, Kuroki H, Kanno H, et al. Haemophilus influenzae infection and Guillain-Barré syndrome. Brain. 2000;123(Pt 10):2171–8. 11004133 10.1093/brain/123.10.2171

[12] Grimaldi-Bensouda L, Alpérovitch A, Besson G, Vial C, Cuisset JM, Papeix C, et al. Guillain-Barre syndrome, influenzalike illnesses, and influenza vaccination during seasons with and without Circulating A/H1N1 viruses. Am J Epidemiol. 2011;174(3):326–35. 21652600 10.1093/aje/kwr072

[13] van den Berg B, van der Eijk AA, Pas SD, Hunter JG, Madden RG, Tio-Gillen AP, et al. Guillain-Barré syndrome associated with preceding hepatitis E virus infection. Neurology. 2014;82(6):491–7. 24415572 10.1212/WNL.0000000000000111

[14] Cao-Lormeau VM, Blake A, Mons S, Lastère S, Roche C, Vanhomwegen J, et al. Guillain-Barré syndrome outbreak associated with Zika virus infection in French Polynesia: a case-control study. Lancet. 2016;387(10027):1531–9. 26948433 10.1016/S0140-6736(16)00562-6 PMC5444521

[15] Stegmann-Planchard S, Gallian P, Tressières B, Leparc-Goffart I, Lannuzel A, Signaté A, et al. Chikungunya, a risk factor for Guillain-Barré syndrome. Clin Infect Dis. 2020;70(6):1233–5. 31290540 10.1093/cid/ciz625

[16] Frontera JA, Tamborska AA, Doheim MF, Garcia-Azorin D, Gezegen H, Guekht A et al. Neurological Events Reported after COVID-19 Vaccines: An Analysis of VAERS. Ann Neurol. 2022. 10.1002/ana.26339 PMC9082459 35233819

[17] Kao JC, Liao B, Markovic SN, Klein CJ, Naddaf E, Staff NP, et al. Neurological complications associated with Anti-Programmed death 1 (PD-1) antibodies. JAMA Neurol. 2017;74(10):1216–22. 28873125 10.1001/jamaneurol.2017.1912 PMC5710300

[18] Pappa E, Berzero G, Herlin B, Ricard D, Tafani C, Devic P, et al. Guillain-Barré syndrome during Platinum-Based chemotherapy: A case series and review of the literature. Oncologist. 2020;25(1):e194–7. 31615948 10.1634/theoncologist.2019-0255 PMC6964130

[19] Rudant J, Dupont A, Mikaeloff Y, Bolgert F, Coste J, Weill A. Surgery and risk of Guillain-Barré syndrome: A French nationwide epidemiologic study. Neurology. 2018;91(13):e1220–7. 30143563 10.1212/WNL.0000000000006246

[20] Kaida Kichi, Morita D, Kanzaki M, Kamakura K, Motoyoshi K, Hirakawa M, et al. Ganglioside complexes as new target antigens in Guillain-Barré syndrome. Ann Neurol. 2004;56(4):567–71. 15389898 10.1002/ana.20222

[21] Yuki N, Yoshino H, Sato S, Miyatake T. Acute axonal polyneuropathy associated with anti-GM1 antibodies following Campylobacter enteritis. Neurology. 1990;40(12):1900–2. 2247243 10.1212/wnl.40.12.1900

[22] Yuki N, Yoshino H, Sato S, Shinozawa K, Miyatake T. Severe acute axonal form of Guillain-Barré syndrome associated with IgG anti-GD1a antibodies. Muscle Nerve. 1992;15(8):899–903. 1495505 10.1002/mus.880150806

[23] Yuki N, Taki T, Inagaki F, Kasama T, Takahashi M, Saito K, et al. A bacterium lipopolysaccharide that elicits Guillain-Barré syndrome has a GM1 ganglioside-like structure. J Exp Med. 1993;178(5):1771–5. 8228822 10.1084/jem.178.5.1771 PMC2191246

[24] Rees JH, Soudain SE, Gregson NA, Hughes RA. Campylobacter jejuni infection and Guillain-Barré syndrome. N Engl J Med. 1995;333(21):1374–9. 7477117 10.1056/NEJM199511233332102

[25] Kusunoki S, Shiina M, Kanazawa I. Anti-Gal-C antibodies in GBS subsequent to Mycoplasma infection: evidence of molecular mimicry. Neurology. 2001;57(4):736–8. 11524496 10.1212/wnl.57.4.736

[26] Wanschitz J, Maier H, Lassmann H, Budka H, Berger T. Distinct time pattern of complement activation and cytotoxic T cell response in Guillain-Barré syndrome. Brain. 2003;126(Pt 9):2034–42. 12847075 10.1093/brain/awg207

[27] Koike H, Katsuno M. Macrophages and autoantibodies in demyelinating diseases. Cells. 2021;10(4):844. 33917929 10.3390/cells10040844 PMC8068327

[28] Kokubun N, Nishibayashi M, Uncini A, Odaka M, Hirata K, Yuki N. Conduction block in acute motor axonal neuropathy. Brain. 2010;133(10):2897–908. 20855419 10.1093/brain/awq260

[29] Vucic S, Cairns KD, Black KR, Chong PST, Cros D. Neurophysiologic findings in early acute inflammatory demyelinating polyradiculoneuropathy. Clin Neurophysiol. 2004;115(10):2329–35. 15351375 10.1016/j.clinph.2004.05.009

[30] Cutillo G, Saariaho AH, Meri S. Physiology of gangliosides and the role of antiganglioside antibodies in human diseases. Cell Mol Immunol. 2020;17(4):313–22. 32152553 10.1038/s41423-020-0388-9 PMC7109116

[31] Blum S, McCombe PA. Genetics of Guillain-Barré syndrome (GBS) and chronic inflammatory demyelinating polyradiculoneuropathy (CIDP): current knowledge and future directions. J Peripher Nerv Syst. 2014;19(2):88–103. 25039604 10.1111/jns5.12074

[32] Geleijns K, Brouwer BA, Jacobs BC, Houwing-Duistermaat JJ, van Duijn CM, van Doorn PA. The occurrence of Guillain-Barre syndrome within families. Neurology. 2004;63(9):1747–50. 15534275 10.1212/01.wnl.0000143055.09646.31

[33] Khanmohammadi S, Malekpour M, Jabbari P, Rezaei N. Genetic basis of Guillain-Barre syndrome. J Neuroimmunol. 2021;358:577651. 34246981 10.1016/j.jneuroim.2021.577651

[34] Ramos AP, Leonhard SE, Halstead SK, Cuba MA, Castañeda CC, Dioses JA, et al. Guillain-Barré syndrome outbreak in Peru 2019 associated with Campylobacter jejuni infection. Neurol Neuroimmunol Neuroinflamm. 2021;8(2):e952. 33547152 10.1212/NXI.0000000000000952 PMC8057064

[35] Guillain-Barré outbreak in Peru. Available from: https://www.who.int/fr/emergencies/disease-outbreak-news/item/2023-DON477 (accessed August 8, 2024).

[36] Wijdicks EF, Ropper AH. Acute relapsing Guillain-Barré syndrome after long asymptomatic intervals. Arch Neurol. 1990;47(1):82–4. 2294897 10.1001/archneur.1990.00530010104027

[37] Grand’Maison F, Feasby TE, Hahn AF, Koopman WJ. Recurrent Guillain-Barré syndrome. Clinical and laboratory features. Brain. 1992;115(4):1093–106. 1393505 10.1093/brain/115.4.1093

[38] Jamall S, Baker N, Peterson G, Tsao B, Rosenfeld J. Aggressive acquired demyelinating neuropathy caused by NF-155: initially Treatment-Resistant. J Clin Neuromuscul Dis. 2023;25(2):59–62. 37962191 10.1097/CND.0000000000000464

[39] Appeltshauser L, Junghof H, Messinger J, Linke J, Haarmann A, Ayzenberg I, et al. Anti-pan-neurofascin antibodies induce subclass-related complement activation and nodo-paranodal damage. Brain. 2023;146(5):1932–49. 36346134 10.1093/brain/awac418 PMC10151189

[40] Devaux JJ, Odaka M, Yuki N. Nodal proteins are target antigens in Guillain-Barré syndrome. J Peripher Nerv Syst. 2012;17(1):62–71. 22462667 10.1111/j.1529-8027.2012.00372.x

[41] Pascual-Goñi E, Caballero-Ávila M, Querol L. Antibodies in autoimmune neuropathies: what to test, how to test, why to test. Neurology. 2024;103(4):e209725. 39088795 10.1212/WNL.0000000000209725 PMC11319070

[42] van Doorn PA, Van den Bergh PYK, Hadden RDM, Avau B, Vankrunkelsven P, Attarian S, et al. European academy of neurology/peripheral nerve society guideline on diagnosis and treatment of Guillain-Barré syndrome. Eur J Neurol. 2023;30(12):3646–74. 37814552 10.1111/ene.16073

[43] Rajabally YA, Uncini A. Outcome and its predictors in Guillain-Barre syndrome. J Neurol Neurosurg Psychiatry. 2012;83(7):711–8. 22566597 10.1136/jnnp-2011-301882

[44] Le Guennec L, Brisset M, Viala K, Essardy F, Maisonobe T, Rohaut B, et al. Post-traumatic stress symptoms in Guillain-Barré syndrome patients after prolonged mechanical ventilation in ICU: a preliminary report. J Peripher Nerv Syst. 2014;19(3):218–23. 25403788 10.1111/jns.12087

[45] Ropper AH. The Guillain-Barré syndrome. N Engl J Med. 1992;326(17):1130–6. 1552914 10.1056/NEJM199204233261706

[46] Leonhard SE, Mandarakas MR, Gondim FAA, Bateman K, Ferreira MLB, Cornblath DR, et al. Diagnosis and management of Guillain-Barré syndrome in ten steps. Nat Rev Neurol. 2019;15(11):671–83. 31541214 10.1038/s41582-019-0250-9 PMC6821638

[47] Ruts L, van Koningsveld R, van Doorn PA. Distinguishing acute-onset CIDP from Guillain-Barré syndrome with treatment related fluctuations. Neurology. 2005;65(1):138–40. 16009902 10.1212/01.wnl.0000167549.09664.b8

[48] Zaeem Z, Siddiqi ZA, Zochodne DW. Autonomic involvement in Guillain-Barré syndrome: an update. Clin Auton Res. 2019;29(3):289–99. 30019292 10.1007/s10286-018-0542-y

[49] Chakraborty T, Kramer CL, Wijdicks EFM, Rabinstein AA. Dysautonomia in Guillain-Barré syndrome: prevalence, clinical spectrum, and outcomes. Neurocrit Care. 2020;32(1):113–20. 31297663 10.1007/s12028-019-00781-w

[50] Ruts L, Drenthen J, Jongen JLM, Hop WCJ, Visser GH, Jacobs BC, et al. Pain in Guillain-Barre syndrome: a long-term follow-up study. Neurology. 2010;75(16):1439–47. 20861454 10.1212/WNL.0b013e3181f88345

[51] Korinthenberg R, Eckenweiler M, Fuchs H. Severe Locked-In-Like Guillain-Barré’s syndrome: dilemmas in diagnosis and treatment. Neuropediatrics. 2021;52(1):19–26. 33111302 10.1055/s-0040-1715480

[52] Faugeras F, Rohaut B, Weiss N, Bekinschtein T, Galanaud D, Puybasset L, et al. Event related potentials elicited by violations of auditory regularities in patients with impaired consciousness. Neuropsychologia. 2012;50(3):403–18. 22230230 10.1016/j.neuropsychologia.2011.12.015

[53] Yuki N, Kokubun N, Kuwabara S, Sekiguchi Y, Ito M, Odaka M, et al. Guillain-Barré syndrome associated with normal or exaggerated tendon reflexes. J Neurol. 2012;259(6):1181–90. 22143612 10.1007/s00415-011-6330-4

[54] Wakerley BR, Soon D, Chan YC, Yuki N. Atypical Bickerstaff brainstem encephalitis: ataxic hypersomnolence without ophthalmoplegia. J Neurol Neurosurg Psychiatry. 2013;84(11):1206–7. 23564757 10.1136/jnnp-2013-304993

[55] Wakerley BR, Yuki N. Polyneuritis cranialis–subtype of Guillain-Barré syndrome? Nat Rev Neurol. 2015;11(11):664. 26369515 10.1038/nrneurol.2015.115

[56] Wakerley BR, Yuki N. Isolated facial diplegia in Guillain-Barré syndrome: bifacial weakness with paresthesias. Muscle Nerve. 2015;52(6):927–32. 26315943 10.1002/mus.24887

[57] Wakerley BR, Yuki N. Pharyngeal-cervical-brachial variant of Guillain-Barre syndrome. J Neurol Neurosurg Psychiatry. 2014;85(3):339–44. 23804237 10.1136/jnnp-2013-305397

[58] Kimachi T, Yuki N, Kokubun N, Yamaguchi S, Wakerley BR. Paraparetic Guillain-Barré syndrome: nondemyelinating reversible conduction failure restricted to the lower limbs. Muscle Nerve. 2017;55(2):281–5. 27397635 10.1002/mus.25242

[59] Wakerley BR, Uncini A, Yuki N, GBS Classification Group, GBS Classification Group. Guillain-Barré and miller fisher syndromes–new diagnostic classification. Nat Rev Neurol. 2014;10(9):537–44. 25072194 10.1038/nrneurol.2014.138

[60] Wakerley BR, Yuki N. Mimics and chameleons in Guillain-Barré and miller fisher syndromes. Pract Neurol. 2015;15(2):90–9. 25239628 10.1136/practneurol-2014-000937

[61] Kuwabara S, Misawa S, Mori M. Is ‘bickerstaff brainstem encephalitis’ really encephalitis? J Neurol Neurosurg Psychiatry. 2013;84(7):712. 23606734 10.1136/jnnp-2012-304655

[62] Criteria for diagnosis of Guillain-Barré syndrome. Ann Neurol. 1978;3(6):565–6. 677829 10.1002/ana.410030628

[63] Asbury AK, Cornblath DR, Assessment of current diagnostic criteria for Guillain-Barré syndrome. Ann Neurol. 1990;27Suppl:S21–24. 10.1002/ana.410270707 2194422

[64] Hadden RD, Cornblath DR, Hughes RA, Zielasek J, Hartung HP, Toyka KV, et al. Electrophysiological classification of Guillain-Barré syndrome: clinical associations and outcome. Plasma Exchange/Sandoglobulin Guillain-Barré syndrome trial group. Ann Neurol. 1998;44(5):780–8. 9818934 10.1002/ana.410440512

[65] Arends S, Drenthen J, van den Bergh P, Franssen H, Hadden RDM, Islam B, et al. Electrodiagnosis of Guillain-Barre syndrome in the international GBS outcome study: differences in methods and reference values. Clin Neurophysiol. 2022;138:231–40. 35078730 10.1016/j.clinph.2021.12.014

[66] Rajabally YA, Durand MC, Mitchell J, Orlikowski D, Nicolas G. Electrophysiological diagnosis of Guillain-Barré syndrome subtype: could a single study suffice? J Neurol Neurosurg Psychiatry. 2015;86(1):115–9. 24816419 10.1136/jnnp-2014-307815

[67] Fortanier E, Berling E, Zanin A, Guillou AL, Micaleff J, Nicolas G, et al. How to distinguish Guillain-Barré syndrome from nitrous oxide-induced neuropathy: A 2-year, multicentric, retrospective study. Eur J Neurol. 2023;30(10):3296–306. 37494104 10.1111/ene.15998

[68] Dawudi Y, Azoyan L, Broucker TDE, Gendre T, Miloudi A, Echaniz-Laguna A, et al. Marked increase in severe neurological disorders after nitrous oxide abuse: a retrospective study in the greater Paris area. J Neurol. 2024;271(6):3340–6. 38478030 10.1007/s00415-024-12264-w PMC11136741

[69] Grimm A, Décard BF, Schramm A, Pröbstel AK, Rasenack M, Axer H, et al. Ultrasound and electrophysiologic findings in patients with Guillain-Barré syndrome at disease onset and over a period of six months. Clin Neurophysiol. 2016;127(2):1657–63. 26220732 10.1016/j.clinph.2015.06.032

[70] Razali SNO, Arumugam T, Yuki N, Rozalli FI, Goh KJ, Shahrizaila N. Serial peripheral nerve ultrasound in Guillain-Barré syndrome. Clin Neurophysiol. 2016;127(2):1652–6. 26228791 10.1016/j.clinph.2015.06.030

[71] Altmann P, De Simoni D, Kaider A, Ludwig B, Rath J, Leutmezer F, et al. Increased serum neurofilament light chain concentration indicates poor outcome in Guillain-Barré syndrome. J Neuroinflammation. 2020;17(1):86. 32183837 10.1186/s12974-020-01737-0 PMC7079539

[72] Martín-Aguilar L, Camps-Renom P, Lleixà C, Pascual-Goñi E, Díaz-Manera J, Rojas-García R et al. Serum neurofilament light chain predicts long-term prognosis in Guillain-Barré syndrome patients. J Neurol Neurosurg Psychiatry. 2020;jnnp-2020-323899. 10.1136/jnnp-2020-323899 33154183

[73] Jacobs BC. Neurofilament light chain as biomarker for axonal damage in Guillain-Barré syndrome. J Neurol Neurosurg Psychiatry. 2020;jnnp-2020-324308. 10.1136/jnnp-2020-324308 PMC7803883 33154185

[74] Pfeiffer G, Schiller B, Kruse J, Netzer J. Indicators of Dysautonomia in severe Guillain-Barré syndrome. J Neurol. 1999;246(11):1015–22. 10631632 10.1007/s004150050506

[75] de Jager AE, Sluiter HJ. Clinical signs in severe Guillain-Barré syndrome: analysis of 63 patients. J Neurol Sci. 1991;104(2):143–50. 1940970 10.1016/0022-510x(91)90303-o

[76] Kumar M, Kalita J, Misra UK. Renal salt wasting in Guillain-Barré syndrome. Postgrad Med J. 2019;95(1129):628–9. 31375556 10.1136/postgradmedj-2019-136870

[77] Cooper WC, Green IJ, Wang SP, CEREBRAL SALT-WASTING ASSOCIATED, WITH THE GUILLAIN-BARR’E SYNDROME. Arch Intern Med. 1965;116:113–9. 14338942 10.1001/archinte.1965.03870010115014

[78] Lenhard T, Grimm C, Ringleb PA. Renal salt wasting as part of Dysautonomia in Guillain-Barre syndrome. J Neurol Neurosurg Psychiatry. 2011;82(9):1051–3. 20732865 10.1136/jnnp.2009.192369

[79] Cochen V, Arnulf I, Demeret S, Neulat ML, Gourlet V, Drouot X, et al. Vivid Dreams, hallucinations, psychosis and REM sleep in Guillain-Barré syndrome. Brain. 2005;128(Pt 11):2535–45. 16000335 10.1093/brain/awh585

[80] Chevret S, Hughes RA, Annane D. Plasma exchange for Guillain-Barré syndrome. Cochrane Database Syst Rev. 2017;2:CD001798. 28241090 10.1002/14651858.CD001798.pub3 PMC6464100

[81] Hughes RAC, Swan AV, van Doorn PA. Intravenous Immunoglobulin for Guillain-Barré syndrome. Cochrane Database Syst Rev. 2014;(9):CD002063. 10.1002/14651858.CD002063.pub6 PMC6781841 25238327

[82] Hughes RA, Brassington R, Gunn AA, van Doorn PA. Corticosteroids for Guillain-Barré syndrome. Cochrane Database Syst Rev. 2016;10:CD001446. 27775812 10.1002/14651858.CD001446.pub5 PMC6464149

[83] Appropriate number of plasma exchanges in Guillain-Barré syndrome. The French cooperative group on plasma exchange in Guillain-Barré syndrome. Ann Neurol. 1997;41(3):298–306. 9066350 10.1002/ana.410410304

[84] Bouget J, Chevret S, Chastang C, Raphael JC. Plasma exchange morbidity in Guillain-Barré syndrome: results from the French prospective, randomized, multicenter study. The French cooperative group. Crit Care Med. 1993;21(5):651–8. 8482086 10.1097/00003246-199305000-00006

[85] Korinthenberg R, Schessl J, Kirschner J, Mönting JS. Intravenously administered Immunoglobulin in the treatment of childhood Guillain-Barré syndrome: a randomized trial. Pediatrics. 2005;116(1):8–14. 15995024 10.1542/peds.2004-1324

[86] Walgaard C, Jacobs BC, Lingsma HF, Steyerberg EW, van den Berg B, Doets AY, et al. Second intravenous Immunoglobulin dose in patients with Guillain-Barré syndrome with poor prognosis (SID-GBS): a double-blind, randomised, placebo-controlled trial. Lancet Neurol. 2021;20(4):275–83. 33743237 10.1016/S1474-4422(20)30494-4

[87] Willison H, Scherer SS. Ranvier revisited: novel nodal antigens stimulate interest in GBS pathogenesis. Neurology. 2014;83(2):106–8. 24920859 10.1212/WNL.0000000000000581

[88] Savithri Nandeesha S, Kasagga A, Hawrami C, Ricci E, Hailu KT, Salib K, et al. Treatment efficacy of plasmapheresis versus intravenous Immunoglobulin in Guillain-Barré syndrome management: A systematic review. Cureus. 2024;16(3):e57066. 38681292 10.7759/cureus.57066 PMC11052558

[89] Randomised trial of. Plasma exchange, intravenous Immunoglobulin, and combined treatments in Guillain-Barré syndrome. Plasma Exchange/Sandoglobulin Guillain-Barré syndrome trial group. Lancet. 1997;349(9047):225–30. 9014908

[90] Kleyweg RP, van der Meché FG. Treatment related fluctuations in Guillain-Barré syndrome after high-dose Immunoglobulins or plasma-exchange. J Neurol Neurosurg Psychiatry. 1991;54(11):957–60. 1800666 10.1136/jnnp.54.11.957 PMC1014615

[91] Lawn ND, Fletcher DD, Henderson RD, Wolter TD, Wijdicks EF. Anticipating mechanical ventilation in Guillain-Barré syndrome. Arch Neurol. 2001;58(6):893–8. 11405803 10.1001/archneur.58.6.893

[92] Kannan Kanikannan MA, Durga P, Venigalla NK, Kandadai RM, Jabeen SA, Borgohain R. Simple bedside predictors of mechanical ventilation in patients with Guillain-Barre syndrome. J Crit Care. 2014;29(2):219–23. 24378177 10.1016/j.jcrc.2013.10.026

[93] Green C, Baker T, Subramaniam A. Predictors of respiratory failure in patients with Guillain-Barré syndrome: a systematic review and meta-analysis. Med J Aust. 2018;208(4):181–8. 29490222 10.5694/mja17.00552

[94] Melone MA, Heming N, Meng P, Mompoint D, Aboab J, Clair B, et al. Early mechanical ventilation in patients with Guillain-Barré syndrome at high risk of respiratory failure: a randomized trial. Ann Intensive Care. 2020;10(1):128. 32997260 10.1186/s13613-020-00742-z PMC7525233

[95] Sharshar T, Chevret S, Bourdain F, Raphaël JC. French cooperative group on plasma exchange in Guillain-Barré syndrome. Early predictors of mechanical ventilation in Guillain-Barré syndrome. Crit Care Med. 2003;31(1):278–83. 12545029 10.1097/00003246-200301000-00044

[96] Walgaard C, Lingsma HF, Ruts L, Drenthen J, van Koningsveld R, Garssen MJP, et al. Prediction of respiratory insufficiency in Guillain-Barré syndrome. Ann Neurol. 2010;67(6):781–7. 20517939 10.1002/ana.21976

[97] Luijten LWG, Doets AY, Arends S, Dimachkie MM, Gorson KC, Islam B, et al. Modified Erasmus GBS respiratory insufficiency score: a simplified clinical tool to predict the risk of mechanical ventilation in Guillain-Barré syndrome. J Neurol Neurosurg Psychiatry. 2023;94(4):300–8. 36428088 10.1136/jnnp-2022-329937

[98] Doets AY, Walgaard C, Lingsma HF, Islam B, Papri N, Yamagishi Y, et al. International validation of the Erasmus Guillain-Barré syndrome respiratory insufficiency score. Ann Neurol. 2022;91(4):521–31. 35106830 10.1002/ana.26312 PMC9306880

[99] Durand MC, Porcher R, Orlikowski D, Aboab J, Devaux C, Clair B, et al. Clinical and electrophysiological predictors of respiratory failure in Guillain-Barré syndrome: a prospective study. Lancet Neurol. 2006;5(12):1021–8. 17110282 10.1016/S1474-4422(06)70603-2

[100] Orlikowski D, Prigent H, Sharshar T, Lofaso F, Raphael JC. Respiratory dysfunction in Guillain-Barré syndrome. Neurocrit Care. 2004;1(4):415–22. 16174943 10.1385/NCC:1:4:415

[101] Chevrolet JC, Deléamont P. Repeated vital capacity measurements as predictive parameters for mechanical ventilation need and weaning success in the Guillain-Barré syndrome. Am Rev Respir Dis. 1991;144(4):814–8. 1928954 10.1164/ajrccm/144.4.814

[102] Kalita J, Kumar M, Misra UK. Serial single breath count is a reliable tool for monitoring respiratory functions in Guillain-Barré syndrome. J Clin Neurosci. 2020;72:50–6. 31982274 10.1016/j.jocn.2020.01.032

[103] Fourrier F, Robriquet L, Hurtevent JF, Spagnolo S. A simple functional marker to predict the need for prolonged mechanical ventilation in patients with Guillain-Barré syndrome. Crit Care. 2011;15(1):R65. 21338488 10.1186/cc10043 PMC3221998

[104] Lawn ND, Wijdicks EF. Tracheostomy in Guillain-Barré syndrome. Muscle Nerve. 1999;22(8):1058–62. 10417787 10.1002/(sici)1097-4598(199908)22:8<1058::aid-mus8>3.0.co;2-g

[105] Schröder JB, Marian T, Muhle P, Claus I, Thomas C, Ruck T, et al. Intubation, tracheostomy, and decannulation in patients with Guillain-Barré-syndrome-does dysphagia matter? Muscle Nerve. 2019;59(2):194–200. 30390307 10.1002/mus.26377

[106] Galassi G, Mazzoli M, Ariatti A, Bedin R, Marzullo D, Bastia E, et al. Predictors of respiratory failure in Guillain-Barré syndrome: a 22 year cohort study from a single Italian centre. Eur J Neurol. 2024;31(1):e16090. 37823704 10.1111/ene.16090 PMC11235807

[107] Cheng MCF, Murphy PB, Hart N, Evans MRB, Spillane JE, Howard RS. Prolonged ventilatory support for patients recovering from Guillain-Barré syndrome. Neurol Clin Pract. 2021;11(1):18–24. 33968468 10.1212/CPJ.0000000000000793 PMC8101308

[108] Nguyen TN, Badjatia N, Malhotra A, Gibbons FK, Qureshi MM, Greenberg SA. Factors predicting extubation success in patients with Guillain-Barré syndrome. Neurocrit Care. 2006;5(3):230–4. 17290095 10.1385/NCC:5:3:230 PMC3817284

[109] Liu J, Wang LN, McNicol ED. Pharmacological treatment for pain in Guillain-Barré syndrome. Cochrane Database Syst Rev. 2015;4CD009950. 10.1002/14651858.CD009950.pub3 PMC6361619 25855461

[110] Gales A, Chaaban B, Husson H, Le Guennec L, Viala K, Maisonobe T, et al. Lidocaine-medicated plaster for treating acute autonomic and sensory neuropathy with erythromelalgia-like presentations. Rev Neurol (Paris). 2016;172(6–7):399–400. 27158041 10.1016/j.neurol.2016.03.004

[111] Doets AY, Hughes RA, Brassington R, Hadden RD, Pritchard J. Pharmacological treatment other than corticosteroids, intravenous Immunoglobulin and plasma exchange for Guillain-Barré syndrome. Cochrane Database Syst Rev. 2020;1:CD008630. 31981368 10.1002/14651858.CD008630.pub5 PMC6984651

[112] Halstead SK, Zitman FMP, Humphreys PD, Greenshields K, Verschuuren JJ, Jacobs BC, et al. Eculizumab prevents anti-ganglioside antibody-mediated neuropathy in a murine model. Brain. 2008;131(Pt 5):1197–208. 18184663 10.1093/brain/awm316

[113] Davidson AI, Halstead SK, Goodfellow JA, Chavada G, Mallik A, Overell J, et al. Inhibition of complement in Guillain-Barré syndrome: the ICA-GBS study. J Peripher Nerv Syst. 2017;22(1):4–12. 27801990 10.1111/jns.12194

[114] Misawa S, Kuwabara S, Sato Y, Yamaguchi N, Nagashima K, Katayama K, et al. Safety and efficacy of Eculizumab in Guillain-Barré syndrome: a multicentre, double-blind, randomised phase 2 trial. Lancet Neurol. 2018;17(6):519–29. 29685815 10.1016/S1474-4422(18)30114-5

[115] Busl KM, Fried H, Muehlschlegel S, Wartenberg KE, Rajajee V, Alexander SA, et al. Guidelines for neuroprognostication in adults with Guillain-Barré syndrome. Neurocrit Care. 2023;38(3):564–83. 36964442 10.1007/s12028-023-01707-3 PMC10241707

[116] Miller RG, Peterson GW, Daube JR, Albers JW. Prognostic value of electrodiagnosis in Guillain-Barré syndrome. Muscle Nerve. 1988;11(7):769–74. 3405243 10.1002/mus.880110714

[117] McKhann GM, Griffin JW, Cornblath DR, Mellits ED, Fisher RS, Quaskey SA. Plasmapheresis and Guillain-Barré syndrome: analysis of prognostic factors and the effect of plasmapheresis. Ann Neurol. 1988;23(4):347–53. 3382169 10.1002/ana.410230406

[118] Walgaard C, Lingsma HF, Ruts L, van Doorn PA, Steyerberg EW, Jacobs BC. Early recognition of poor prognosis in Guillain-Barre syndrome. Neurology. 2011;76(11):968–75. 21403108 10.1212/WNL.0b013e3182104407 PMC3059137

[119] Walgaard C, Lingsma HF, van Doorn PA, van der Jagt M, Steyerberg EW, Jacobs BC. Tracheostomy or not: prediction of prolonged mechanical ventilation in Guillain-Barré syndrome. Neurocrit Care. 2017;26(1):6–13. 27538676 10.1007/s12028-016-0311-5 PMC5227005

[120] Terayama A, Kuwahara M, Yoshikawa K, Yamagishi Y, Samukawa M, Yamashita S, Onishi K, Nagano T, Tatsumi C, Ishii J, Kawamoto M, Tokashiki T, Deguchi S, Deguchi K, Ishida A, Baba Y, Yamaguchi S, Kusunoki S, Nagai Y. Takotsubo cardiomyopathy in Guillain-Barré syndrome. J Neurol. 2024;271(7):4067–74. 38573364 10.1007/s00415-024-12295-3

[121] Sharshar T, Polito A, Porcher R, Merhbene T, Blanc M, Antona M, Durand MC, Friedman D, Orlikowski D, Annane D, Marcadet MH. Relevance of anxiety in clinical practice of Guillain-Barre syndrome: a cohort study. BMJ Open. 2012;2(4):e000893. 10.1136/bmjopen-2012-000893 PMC3432836 22923622

[122] Polito A, Polito A, Bouchereau E, Moneger G, Ritzenthaler T, Annane D, Heming N, Sharshar T. Dysglycemia and neurologic outcome in mechanically ventilated patients with Guillain-Barré syndrome. Crit Care Med. 2019;47(3):e227–33. 30585828 10.1097/CCM.0000000000003635

[123] Khan F, Amatya B. Rehabilitation interventions in patients with acute demyelinating inflammatory polyneuropathy: a systematic review. Eur J Phys Rehabil Med. 2012;48:507–22. 22820829

[124] Simatos Arsenault N, Vincent PO, Yu BH, Bastien R, Sweeney A. Influence of exercise on patients with Guillain-Barré syndrome: A systematic review. Physiother Can. 2016;68(4):367–76. 27904236 10.3138/ptc.2015-58 PMC5125499

[125] Kuwabara S, Kusunoki S, Kuwahara M, Yamano Y, Nishida Y, Ishida H, Kasuya T, Kupperman E, Lin Q, Frick G, Misawa S. Efficacy and safety of Eculizumab in Guillain-Barre syndrome: A phase 3, multicenter, double-blind, randomized, placebo-controlled clinical trial. J Peripher Nerv Syst. 2024;29(3):339–49. 38987228 10.1111/jns.12646

[126] Melone MA, Heming N, Meng P, Mompoint D, Aboab J, Clair B, Salomon J, Sharshar T, Orlikowski D, Chevret S, Annane D. Early mechanical ventilation in patients with Guillain-Barré syndrome at high risk of respiratory failure: a randomized trial. Ann Intensive Care. 2020;10(1):128. 32997260 10.1186/s13613-020-00742-z PMC7525233

[127] Orlikowski D, Sharshar T, Porcher R, Annane D, Raphael JC, Clair B. Prognosis and risk factors of early onset pneumonia in ventilated patients with Guillain-Barré syndrome. Intensive Care Med. 2006;32(12):1962–9. 17019557 10.1007/s00134-006-0332-1

[128] Dhar R, Stitt L, Hahn AF. (2008) The morbidity and outcome of patients with Guillain-Barré syndrome admitted to the intensive care unit. J Neurol Sci 2008, 264:121–128. 10.1016/j.jns.2007.08.005 17881005

[129] Bernardes Neto SCG, Torres-Castro R, Lima Í, Resqueti VR, Fregonezi GAF. Weaning from mechanical ventilation in people with neuromuscular disease: a systematic review. BMJ Open. 2021;11(9):e047449. 10.1136/bmjopen-2020-047449 PMC8442075 34521661

[130] Orlikowski D, Prigent H, Raphaël JC, Sharshar T. Acute respiratory failure in Guillain-Barré syndrome and myasthenia gravis. Réanimation 2005 14(2):118–125.

[131] Mazeraud A, Sivanandamoorthy S, Mancusi R, Clair B, Friedman D, Fadel F, Maxime V, Legouy C, Orlikowski D, Sharshar T, Heming N, Annane D. Weaning from mechanical ventilation during myasthenic crisis, a monocentric retrospective study. Sci Rep. 2024;14(1):19523. 39174610 10.1038/s41598-024-70373-y PMC11341545

[132] Mezidi M, Yonis H, Chauvelot L, Deniel G, Dhelft F, Gaillet M, Noirot I, Folliet L, Chabert P, David G, Danjou W, Baboi L, Bettinger C, Bernon P, Girard M, Provoost J, Bazzani A, Bitker L, Richard JC. Spontaneous breathing trial with pressure support on positive end-expiratory pressure and extensive use of non-invasive ventilation versus T-piece in difficult-to-wean patients from mechanical ventilation: a randomized controlled trial. Ann Intensive Care. 2024;14(1):59. 38630372 10.1186/s13613-024-01290-6 PMC11024068

[133] Egger M, Finsterhölzl M, Farabegoli D, Wippenbeck F, Schlutt M, Müller F, Huge V, Jahn K, Bergmann J. Comprehensive assessment and progression of health status during neurorehabilitation in survivors of critical illness: a prospective cohortstudy. Ann Intensive Care. 2024;14(1):175. 39589665 10.1186/s13613-024-01396-x PMC11599680

